# Rapid Response of Advanced Squamous Non-Small Cell Lung Cancer with Thrombocytopenia after First-Line Treatment with Pembrolizumab Plus Autologous Cytokine-Induced Killer Cells

**DOI:** 10.3389/fimmu.2015.00633

**Published:** 2015-12-17

**Authors:** Zhenzhen Hui, Xinwei Zhang, Baozhu Ren, Runmei Li, Xiubao Ren

**Affiliations:** ^1^Department of Biotherapy, Tianjin Medical University Cancer Institute and Hospital, Tianjin, China; ^2^National Clinical Research Center for Cancer, Tianjin, China; ^3^Key Laboratory of Cancer Immunology and Biotherapy, Tianjin, China; ^4^Key Laboratory of Cancer Prevention and Therapy, Tianjin, China; ^5^Department of Immunology, Tianjin Medical University Cancer Institute and Hospital, Tianjin, China

**Keywords:** non-small cell lung cancer, pembrolizumab, cytokine-induced killer cells, thrombocytopenia, immunotherapy

## Abstract

We present the first clinical evidence of advanced squamous non-small cell lung cancer with severe thrombocytopenia showing dramatic improvement after first-line treatment with pembrolizumab plus autologous cytokine-induced killer cells.

## Introduction

Despite great progress in the management of advanced non-small cell lung cancer (NSCLC), chemotherapy remains the therapeutic mainstay for squamous NSCLC. However, only patients with good performance status and sufficient organ function are likely to benefit from this approach. Immunotherapy is an evolving treatment approach based on the use of the immune system to treat cancer. Programed death 1 (PD-1), functioning as an immune checkpoint, plays an important role in tumor immune escape by preventing the activation of T-cells. Nivolumab, a PD-1 blocking antibody, was recently approved by the US Food and Drug Administration (FDA) for patients with advanced NSCLC whose disease progressed during or after platinum-based chemotherapy (1, 2). Similarly, FDA has granted approval for Keytruda (pembrolizumab) to treat patients with advanced (metastatic) NSCLC whose disease has progressed after other treatments and with tumors that express a protein called PD-L1 (3). However, unfortunately, only a subset of patients will benefit from PD-1 blocking antibody. Although some clinical trials suggest that cytokine-induced killer cell (CIK cell) immunotherapy could improve the clinical outcome of advanced squamous cell NSCLC, the treatment is mainly combined with chemotherapy. A recent study indicated that PD-1 blocking antibody may improve the efficacy of adoptive cell therapy in human cancer (4, 5). We report here the successful management of a case of advanced squamous NSCLC with severe thrombocytopenia, treated with pembrolizumab combined with autologous CIK cells as first-line therapy.

## Case Presentation

**Figure 1:**
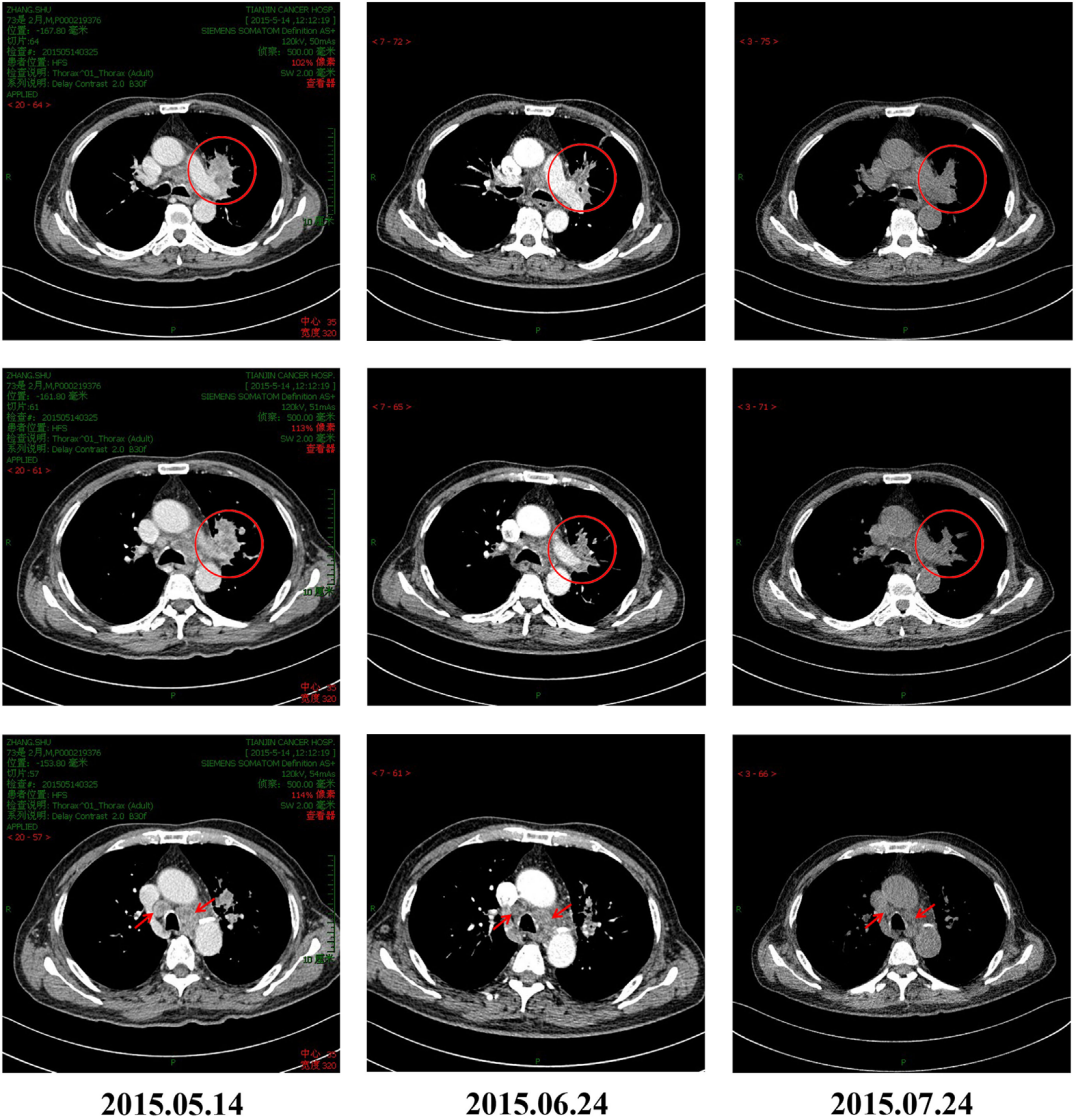
**CT scans of the patient before and after pembrolizumab and CIK combination treatment for advanced NSCLC**. Tumor masses can be seen in the left lung hilum and mediastinal lymph nodes of the patient before initiation of immunotherapy. The masses significantly reduced in size on CT obtained 30 and 60 days after treatment, indicating good response to combination therapy.

**Figure 2:**
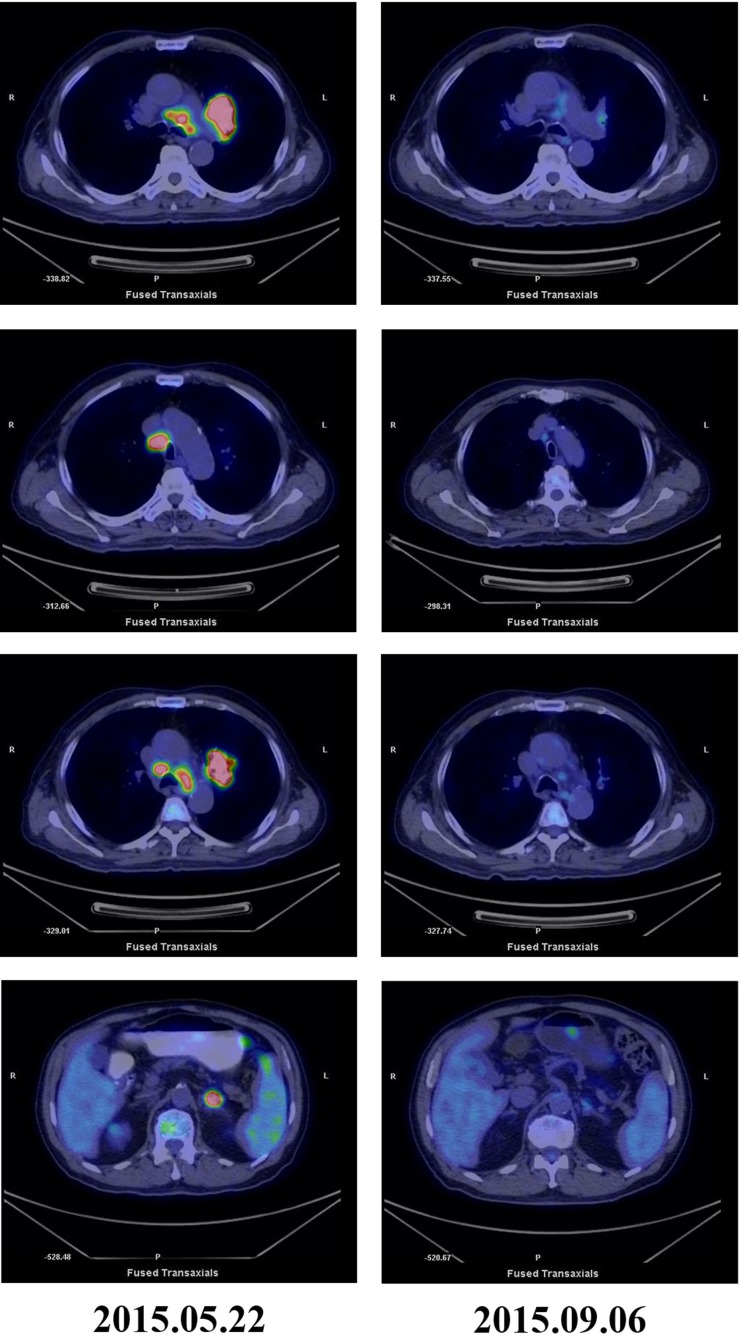
**PET-CT scans before and after treatment**. PET-CT May 2015 before treatment showing high glucose metabolism in left hilar mass, multiple mediastinal lymph nodes, and left adrenal masses. PET-CT September 2015 after five courses of pembrolizumab and three courses of CIK cell therapy showing significantly reduction in tumor mass and FDG uptake.

A 73-year-old man presented at our institution with complaints of fever, cough, and hemoptysis, a computed tomography (CT) and positron emission computed tomography (PET-CT) scan revealed a left hilar mass with multiple pathologically enlarged mediastinal lymph nodes and left adrenal masses (Figures 1 and 2). Bronchoscopy demonstrated an endobronchial tumor obstructing the left bronchus; biopsy revealed poorly differentiated squamous cell carcinoma. Serum tumor markers SCC and Cyfra21-1 were 7.7 and 10.2 μg/L, respectively. Complete blood count analysis showed that the patient had thrombocytopenia (platelet count 5–17 × 10^9^/L). A bone marrow aspirate and PET-CT revealed no abnormalities of bone or bone marrow. The patient was not appropriate for chemo-radiotherapy and at a high risk of massive hemorrhage due to the endobronchial disease coupled with severe thrombocytopenia. Under patient’s informed consent, pembrolizumab combined with autologous CIK cell therapy was initiated as first-line treatment. Meanwhile, written permission from the patient for publication of clinical data was obtained.

**Figure 3:**
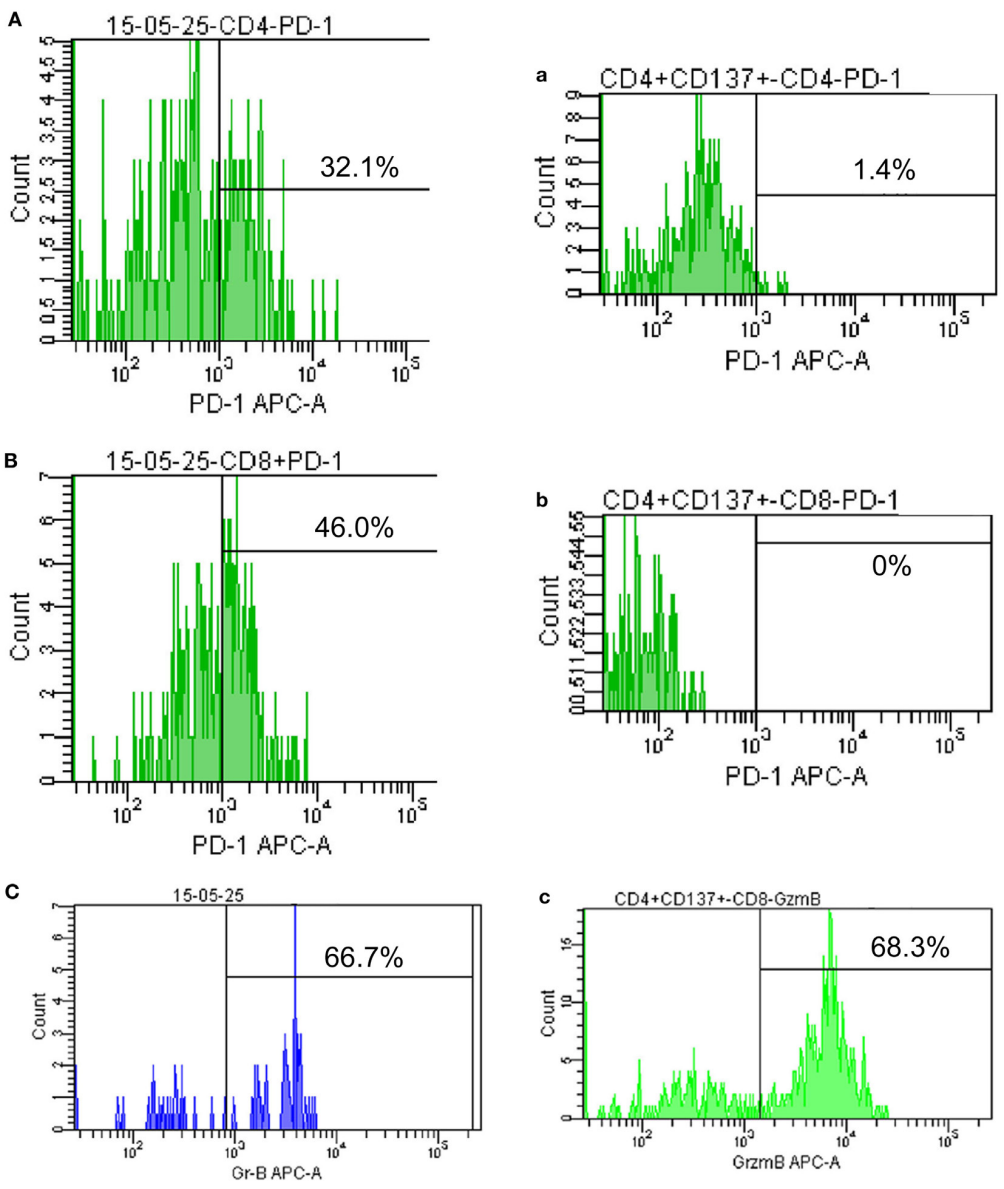
**Programed death 1 and granzyme B expression on peripheral lymphocytes**. **(A–C)** show PD-1 and granzyme B expression on peripheral CD4+ and CD8+ T cells before therapy (May 2015); **(a–c)** show PD-1 and granzyme B expression on CD4+ and CD8+ T cells after achieving PR (September 2015).

Cytokine-induced killer cells are a group of immune effector cells featuring a mixed T- and natural killer (NK) cell-like phenotype, which are generated by incubation of mononuclear cells from peripheral blood with an anti-CD3 monoclonal antibody, interleukin (IL)-2, IL-1, and interferon (IFN)-γ (6, 7). The protocol of CIK preparation was approved by the Institutional Ethics Committee. Peripheral blood mononuclear cells (PBMCs) were collected from patient using a Cobe Spectra Apheresis System (CaridianBCT, Lakewood, CO, USA), and cultured in X-VIVO 20 serum-free medium (Cambrex, East Rutherford, NJ, USA) containing 50 ng/mL anti-CD3 antibody to stimulate CIK cell growth, 100 U/mL recombinant human IL-1a (e-Bioscience, San Diego, CA, USA), and 1,000 U/mL recombinant human IFN-γ (Peprotech, Rocky Hill, NJ, USA), at 37°C with 5% CO_2_ for 24 h. Then, 300 U/mL recombinant human IL-2 (Peprotech) was added to the media. IL-2- and IFN-γ-containing medium was added to the culture system every 5 days. On day 14, CIK cells were harvested and analyzed for phenotype and cytotoxicity. Safety testing was performed during the course of cell culture. All products were free of bacterial and fungal contamination, negative for mycoplasma, and contained <5 Eu endotoxin. Patient was treated with intravenous infusions of (13.07 ± 1.37) × 10^9^ CIK cells at days 15 and 16 of each cycle. These cells have higher proliferation rate, cytolytic activities, and non-MHC-restricted killing of tumor cells (8). After two courses of the immune checkpoint inhibitor (pembrolizumab 2 mg/kg every 3 weeks) and one course of CIK cell therapy (every month), a CT scan revealed a rapid regression in the tumor size (Figure 1). Serum tumor markers also showed a significant decrease: SCC 1.6 μg/L, Cyfra21-1 1.62 μg/L. Another course of pembrolizumab and CIK cell immunotherapy was given and a subsequent CT scan showed further reduction (achieved partial response according to RECIST1.1 criteria) (Figure 1). After five courses of pembrolizumab and three courses of CIK cell therapy, a PET-CT scan showed that both tumor burden and radioactivity were significantly decreased (Figure 2). PD-1 expression on peripheral CD4+ and CD8+ T cells showed dramatically decrease after therapy (32.1 vs.1.4, 46.0 vs. 0%), and there was mild increase in granzyme B expression on CD8+ T cells (66.7 vs. 68.3%) (Figure 3). The patient developed low-grade fatigue, decreased appetite and vitiligo, without serious hemorrhage during treatment, and platelet count rises to 60–144 × 10^9^/L.

## Discussion

Our work represents the first clinical evidence that PD-1 blocking antibody plus autologous CIK cell therapy is well tolerated and highly effective in an advanced squamous NSCLC patient with severe thrombocytopenia. Pembrolizumab works by blocking the PD-1 and keeping its ligands PD-L1 and PD-L2 from binding to it to reduce inhibitory signaling and restore the patient’s natural tumor specific T-cell-mediated immune responses and provide a favorable circumstance for CIK cell infusion. CIK cells could exert a more potent antitumor effect in a tumor microenvironment where the immunoinhibitory effect of PD-L1 is neutralized. The synergistic activity between checkpoint inhibitors and CIK cell infusion could represent an effective therapeutic strategy with rapid onset, which differs from traditional chemotherapy and targeted agents (9). This is only a feasibility case report and our assumption that there is synergy between the antibody and CIK cell is not justified, further research is being carried out in our institution to validate these findings.

## Author Contributions

XR, BR, and RL were responsible for the literature search and proposed treatment strategy. XZ carried out the analysis and interpretation of data. ZH was responsible for data collection, figures and drafted the manuscript. All authors read and approved the final manuscript.

## Conflict of Interest Statement

The authors declare that the research was conducted in the absence of any commercial or financial relationships that could be construed as a potential conflict of interest.
